# Serum Activin A Levels and Renal Outcomes After Coronary Angiography

**DOI:** 10.1038/s41598-020-60359-x

**Published:** 2020-02-25

**Authors:** Yi-Lin Tsai, Ruey-Hsing Chou, Ya-Wen Lu, Chung-te Liu, Po-Hsun Huang, Shing-Jong Lin

**Affiliations:** 1 Division of Cardiology, Department of Medicine, Taipei Veterans General Hospital, Taipei, Taiwan; 2 Cardiovascular Research Center, Taipei Veterans General Hospital, Taipei, Taiwan; 3 Department of Critical Care Medicine, Taipei Veterans General Hospital, Taipei, Taiwan; 4 Institute of Clinical Medicine, National Yang-Ming University, Taipei, Taiwan; 5 Healthcare and Management Center, Taipei Veterans General Hospital, Taipei, Taiwan; 6 Division of Nephrology, Department of Medicine, Wan Fang Hospital, Taipei Medical University, Taipei, Taiwan; 7 School of Medicine, College of Medicine, Taipei Medical University, Taipei, Taiwan; 8 Graduate Institute of Clinical Medicine, College of Medicine, Taipei Medical University, Taipei, Taiwan; 9 Board of Directors, Taipei Medical University, Taipei, Taiwan

**Keywords:** Interventional cardiology, Acute kidney injury

## Abstract

Prevention for contrast-induced nephropathy (CIN) is limited by the lack of a single predictor. As activin A is upregulated in heart failure and chronic kidney disease, we aimed to clarify the association between activin A levels and renal outcomes after coronary angiography (CAG). This prospective observational study included 267 patients who received CAG between 2009 and 2015. CIN was defined as elevation of serum creatinine to >0.5 mg/dL or to >25% above baseline within 48 hours after CAG. During follow-up, laboratory parameters were measured every 3–6 months. Renal decline was defined as>2-fold increase in serum creatinine or initiation of dialysis. The patients were stratified into tertiles according to serum activin A levels at baseline. High activin A tertile was significantly associated more CIN and renal function decline compared to low activin A tertile (all *p* < 0.001). After adjusting potential confounding factors, high serum activin A tertiles was associated to CIN (Odds ratio 4.49, 95% CI 1.07–18.86, *p* = 0.040) and renal function decline (Hazard ratio 4.49, 95% CI 1.27–11.41, *p* = 0.017) after CAG.

## Introduction

Contrast-induced nephropathy (CIN) refers to acute kidney injury (AKI) caused by the contrast medium administered for angiographic procedures, which is one of the major causes of in-hospital AKI^1^. Although CIN is generally transient and reversible, it remains an important clinical issue due to its high incidence and association with adverse outcomes. Previous studies showed that CIN affects 7–25% of patients receiving angiographic procedures^2,3^. Moreover, CIN increases hospitalization duration, medical costs, long-term morbidity, and mortality^4–7^. The clinical significance of CIN is further supported by the fact that even mild elevation of serum creatinine levels after administration of contrast medium is associated with progressive renal decline and development of end-stage renal disease (ESRD)^8^. While it has been accepted that the prevention of CIN is essentially important, the available strategies for treating established CIN remain conservative and limited to fluid and electrolyte management^9^.

Currently, CIN preventive measures start with risk stratification, which involves non-modifiable fixed risk factors, including preexisting chronic kidney disease (CKD), diabetes mellitus, congestive heart failure (CHF), advanced age, female sex, and modifiable risk factors, including hypotension, anemia, concurrent use of nephrotoxic drugs, hypercholesterolemia, dehydration, hyperglycemia, and the type and volume of contrast medium used^10^. For patients at low risk, oral or intravenous hydration are appropriate preventive measures. On the other hand, for patients at intermediate or high risk, correction of the modifiable risk factors should be considered, which include intravenous crystalloid fluid, N-acetylcysteine, ascorbic acid, and statins^10,11^. While the preventive strategy of CIN depends on risk stratification, a single and reliable predictor of CIN remains absent to date.

Activin A is a secreted cytokine of the transforming growth factor-β superfamily first identified in 1986^12–14^. Activin A regulates cell proliferation and differentiation, stimulates inflammation^15^, and inhibits osteogenesis^16^. In addition, activin A activates fibroblasts in various tissues, particularly in kidneys^17,18^, which explains its association with renal fibrosis and progression of CKD^19–22^. Activin receptors are upregulated in the skeleton, vasculature, heart, and kidneys of CKD patients^23^. Increased activin A expression was also found in animal models of renal ischemia/reperfusion^24,25^. These previous findings support a potential role of activin A in AKI and its possible association with CIN or progressive renal decline. On the other hand, elevated serum activin A levels were reported in patients with various cardiovascular diseases including hypertension, atherosclerosis, left ventricular dysfunction, pulmonary hypertension, and CHF^26–29^. Considering the close interaction between heart failure and renal failure^5,8,30–32^, serum activin A may be the ideal biomarker of renal outcomes in patients undergoing coronary angiography (CAG).

As such, while the association between serum activin A levels and post-CAG renal outcomes remains to be defined, we hypothesized that serum activin A levels predict CIN and post-CAG renal function decline. To test this hypothesis, we conducted a prospective cohort study in patients who received CAG to monitor the occurrence of CIN and long-term kidney function.

## Materials and Methods

### Study design and subjects

In this prospective observational study, patients were recruited between December 2009 and February 2015 at Taipei Veterans General Hospital in Taipei, Taiwan. The eligibility screen included 540 patients who presented to the outpatient department with angina and were scheduled for CAG. The exclusion criteria for this study were age <18 years, pre-procedural estimated glomerular filtration rate (eGFR) <15 mL/min/1.73 m^2^, and pre-existing ESRD. The present analysis excluded 32 patients with eGFR <15 mL/min/1.73 m^2^ or pre-existing ESRD, as well as 241 patients lost to follow-up. Overall, 267 patients were included in the present study. All patients with regular follow-up received the measurement of serum creatinine levels and other laboratory parameters every 3–6 months. The patients were carefully monitored for CIN or progressive renal function decline. For statistical analysis, the patients were stratified into three groups based on the tertiles of the distribution of serum activin A levels before CAG. Follow-up data were censored after January 2016, ensuring that all included patients were followed-up for at least one year. All participants provided written informed consent. The study was approved by the research ethics committee of the Taipei Veterans General Hospital (2016-08-002AC) and was conducted in accordance with the tenets of the 1975 Declaration of Helsinki, as revised in 2000.

### Definition of covariates and outcomes

All participants were asked to complete a questionnaire collecting data on daily medication, smoking status, hypertension, diabetes mellitus, and CKD. Afterwards, research nurses conducted anthropometric measurements of height, body weight, blood pressure, and waist circumference. The body mass index (BMI, in kg/m^2^) was calculated by dividing the weight by the height squared. Blood samples were obtained in the morning, after an overnight fast of at least 10 hours. Serum levels of creatinine, glucose, and uric acid were measured using an automatic analyzer (AVDIA 1800; Siemens, Malvern, PA, USA). In the present study, eGFR was calculated using the equation suggested by the Chronic Kidney Disease Epidemiology Collaboration in 2009^33^.

All participants received standard CAG, as well as left ventriculography to evaluate left ventricular ejection fraction (LVEF). Patients with significant coronary artery disease were indicated for standard percutaneous coronary intervention. After the angiographic procedure, serum creatinine levels were followed consecutively for 3 days. The Mehran risk score was calculated to quantify the risk of CIN. Urine dipstick analysis was performed using a commercial test strip, and proteinuria was defined as urine protein levels ≥30 mg/dL. The serum levels of activin A were measured using an enzyme-linked immunosorbent assay kit (Human Activin A Quantikine ELISA Kit, catalog ID DAC00B; R&D Systems, Inc., Minneapolis, MN, USA).

CIN was defined as elevation of serum creatinine levels to >0.5 mg/dL or to >25% above baseline within 48 hours of CAG^4^. The peak creatinine concentration was defined as the highest creatinine concentration recorded within one week after administration of contrast medium. Progressive renal function decline was defined as>2-fold increase in serum creatinine levels or initiation of dialysis treatment during follow-up period^34,35^.

### Statistical analysis

Categorical variables are expressed as frequency (percentage), whereas continuous variables are expressed as mean ± standard deviation. The χ^2^ test was used to compare categorical variables, whereas continuous variables were compared using the Kruskal-Wallis test. The correlation between activin A levels, pre-CAG creatinine level and eGFR were analyzed using the Spearman’s rank correlation coefficient. Logistic regression was used to analyze the association between activin A levels and the risk of CIN. Cox proportional hazard regression analysis and Kaplan-Meier analysis were used to clarify the association between serum activin A levels and progressive renal decline. All statistical analyses were performed using dedicated software (SPSS version 24.0.0.0; IBM Corporation, Armonk, NY, USA). Two-tailed *p*-values <0.05 were considered to indicate statistical significance.

## Results

### Demographic and laboratory characteristics of patients receiving CAG

Among the 267 included patients, the mean age was 71 ± 13 years, and 67% were male. The mean value of serum creatinine was 1.5 mg/dL, eGFR was 61.1 mL/min/1.73 m^2^, and Mehran risk score was 7.1, respectively. Overall, 19.9% of patients had proteinuria, 74.9% had hypertension, 34.5% had diabetes mellitus, and 17.2% had CHF. The mean serum activin A was 562 pg/mL.

**Figure 1:**
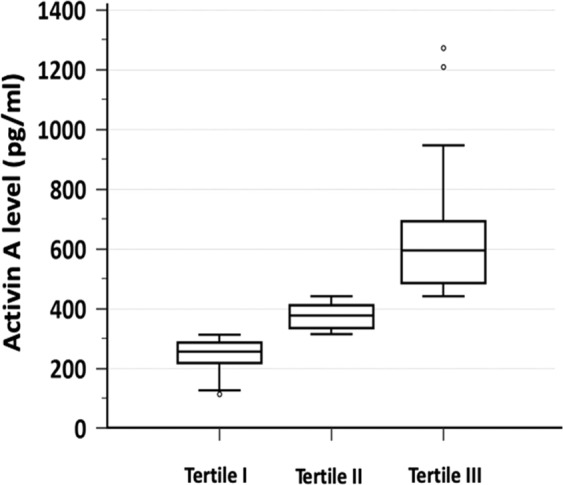
Boxplot of per-CAG serum activin A levels stratified into tertiles. The patients were stratified according to the tertiles of the distribution of serum activin A levels, as follows: tertile I, <312 pg/mL; tertile II, 312–441 pg/mL; tertile III, >441 pg/mL. CAG, coronary angiography.

The participants were stratified into tertiles according to serum activin A levels as following: tertile I, <312 pg/mL (n = 89); tertile II, 312–441 pg/mL (n = 89); tertile III, >441 pg/mL (n = 89) (Fig. 1). Tertile III was characterized by significantly higher serum creatinine level, lower eGFR, lower hemoglobin, lower LVEF and higher Mehran risk score (Table 1). In addition, tertile III exhibited more proteinuria, more hypertension, more smokers, and more use of diuretics. Notably, all the above characters are the suggested risk factors of CIN. Tertile III was also showed trends of higher fasting glucose and more diabetes mellitus, but both did not achieve statistical significance. On the other hand, age, gender, BMI, frequency of CHF and coronary artery disease, and use of angiotensin converting enzyme inhibitors/angiotensin II receptor blockers and statins were not significantly different between the tertiles. Considering that pre-CAG renal function is the most important predictor for CIN, its correlation to Activin A level was examined. It showed that Activin A level was moderately correlated with both creatinine level and eGFR (Table 2). These findings indicated that higher activin A level may have higher risk for CIN.

**Table 1:** Baseline demographic and laboratory characteristics stratified by serum activin A levels.

Characteristic	Total (n = 267)	Tertile I (n = 89)	Tertile II (n = 89)	Tertile III (n = 89)	*p*-value
Age, years	71 ± 13	65 ± 13	76 ± 10	71 ± 14	0.03
Male sex	179 (67%)	60 (67%)	65 (73%)	54 (61%)	0.21
BMI, kg/m^2^	25.6 ± 4.0	25.5 ± 4.2	25.6 ± 3.5	25.5 ± 3.9	0.79
Smoking	98 (37%)	22 (25%)	37 (42%)	39 (44%)	0.02
HTN	200 (75%)	56 (63%)	73 (82%)	71 (80%)	0.006
DM	92 (35%)	25 (28%)	31 (35%)	36 (40%)	0.22
CHF	46 (17%)	13 (15%)	15 (17%)	18 (20%)	0.61
CAD	152 (57%)	50 (56%)	52 (58%)	50 (56%)	0.57
ACEi or ARB	84 (32%)	25 (28%)	29 (33%)	30 (34%)	0.69
Diuretics	46 (17%)	6 (7%)	15 (17%)	25 (28%)	0.001
Statins	62 (23%)	23 (26%)	19 (21%)	20 (23%)	0.76
Cr, mg/dL	1.5 ± 1.5	1.1 ± 0.3	1.2 ± 0.4	2.2 ± 2.4	0.004
eGFR, mL/min/1.73 m^2^	61.1 ± 24.8	73.0 ± 19.1	60.0 ± 19.1	50.2 ± 29.3	<0.001
Proteinuria	53 (20%)	3 (3%)	14 (16%)	36 (40%)	<0.001
Hemoglobin, g/dL	12.5 ± 1.9	13.3 ± 1.7	12.8 ± 1.7	11.7 ± 2.0	<0.001
Fasting glucose, mg/dL	116.3 ± 42.1	111.5 ± 29.0	108.7 ± 26.4	128.8 ± 59.9	0.06
Uric acid, mg/dL	6.4 ± 2.1	5.9 ± 1.7	6.4 ± 2.0	6.9 ± 2.0	0.18
Activin A, pg/mL	562.0 ± 682.5	249.8 ± 47.9	375.6 ± 39.9	627.8 ± 169.5	<0.001
LVEF, %	53.2 ± 11.7	56.9 ± 9.6	54.4 ± 10.7	47.8 ± 12.9	<0.001
Contrast volume, mL	97.6 ± 72.3	102.1 ± 77.3	92.9 ± 65.1	97.8 ± 74.7	0.79
Mehran risk score	7.1 ± 5.3	4.7 ± 4.6	7.7 ± 4.8	9.0 ± 5.5	<0.001

Data represent mean ± standard deviation or frequency (percentage). Distribution of serum activin A levels: tertile I, <312 pg/mL; tertile II, 312–441 pg/mL; tertile III, >441 pg/mL. BMI, body mass index; HTN, hypertension; DM, diabetes mellitus; CHF, congestive heart failure; CKD, chronic kidney disease; CAD, coronary artery disease; ACEi, angiotensin converting enzyme inhibitor; ARB, angiotensin II receptor blocker; Cr, creatinine; eGFR, estimated glomerular filtration rate; LVEF, left ventricular ejection fraction.

**Table 2:** Correlation coefficients for activin A and renal function.

Variables	Activin A	Creatinine	eGFR
Activin A	1	0.316**	−0.373**
Creatinine	0.316**	1	−0.992**

**Correlation is significant at the 0.01 level (two-tailed).

*Correlation is significant at the 0.05 level (two-tailed)

eGFR, estimated glomerular filtration rate.

### Pre-CAG serum levels of activin A and renal outcomes after CAG

Among the 267 patients included in the study, the mean peak creatinine concentration during follow-up was 1.6 mg/dL, and 27 (10%) patients developed CIN. The mean duration of follow-up was 2.4 years. By the end of the follow-up period, 44 (16.5%) patients had developed progressive renal decline, and 11 (4.1%) patients had been started on dialysis treatment.

**Figure 2:**
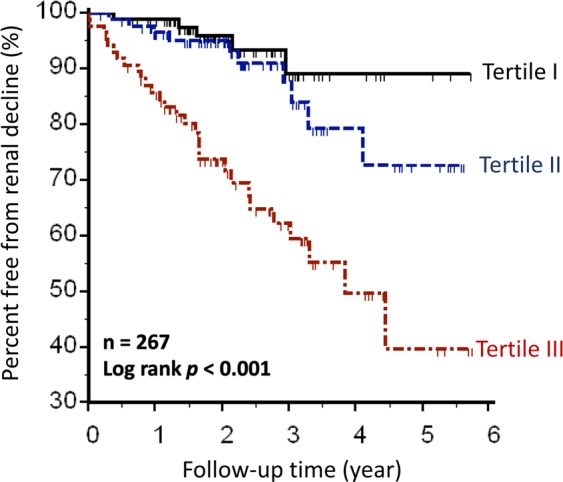
Renal function decline after CAG and its relationship with the serum levels of activin A before CAG. The curves were obtained through Kaplan-Meier analysis and the significance was tested using the log-rank test. The patients were stratified according to the tertiles of the distribution of serum activin A levels, as follows: tertile I, <312 pg/mL; tertile II, 312–441 pg/mL; tertile III, >441 pg/mL. CAG, coronary angiography.

The tertiles did not differ significantly in terms of the contrast medium volumes used in the angiographic procedure. However, the incidence of CIN was significantly higher in tertile III. Additionally, tertile III was characterized by significantly higher peak serum creatinine concentration, higher need for dialysis, and higher incidence of progressive renal function decline during follow-up (Table 3). Kaplan-Meier method revealed that renal function decline during follow-up was the most severe in tertile III, less severe in tertile II, and the least severe in tertile I, with a significant difference on the log-rank test (Fig. 2). These findings indicated that higher pre-procedural serum activin A levels were associated with adverse renal outcomes after CAG.

**Table 3:** Renal outcomes after coronary angiography, stratified by serum activin A levels at baseline.

Characteristic	Total (n = 267)	Tertile I (n = 89)	Tertile II (n = 89)	Tertile III (n = 89)	*p*-value
Peak Cr, mg/dL^‖^	1.6 ± 1.6	1.1 ± 0.4	1.2 ± 0.4	2.4 ± 2.6	<0.001
CIN	27 (10%)	5 (6%)	5 (6%)	17 (19%)	0.003
Dialysis	11 (4%)	0 (0%)	0 (0%)	11 (4%)	<0.001
Renal function decline^*^	44 (17%)	5 (6%)	10 (11%)	29 (33%)	<0.001
Duration of follow-up, years	2.4 ± 1.3	2.3 ± 1.1	2.5 ± 1.3	2.4 ± 1.5	0.46
Contrast volume, mL	97.6 ± 72.3	102.1 ± 77.3	92.9 ± 65.1	97.8 ± 74.7	0.79

Data represent mean ± standard deviation or frequency (percentage). Distribution of serum activin A levels: tertile I, <312 pg/mL; tertile II, 312–441 pg/mL; tertile III, >441 pg/mL. Cr, creatinine; CIN, contrast-induced nephropathy.

^‖^Peak Cr was defined as the highest serum Cr concentration recorded during follow-up.

^*^Renal function decline was defined as >2-fold increase in serum Cr or initiation of dialysis treatment during follow-up.

### Serum activin A levels as predictors of adverse renal outcomes after CAG

**Figure 3:**
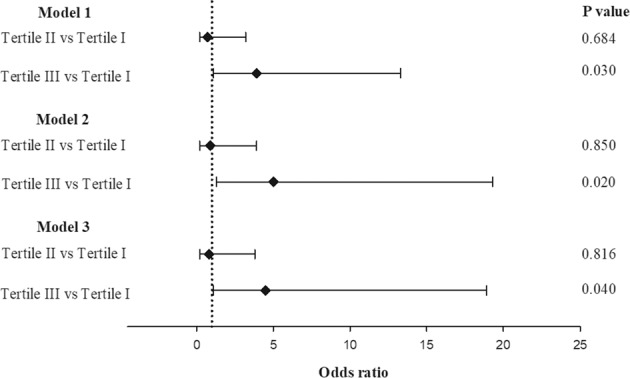
Predictive capability of pre-CAG levels of serum activin A for the risk of contrast-induced nephropathy after CAG. The patients were stratified according to the tertiles of the distribution of activin A levels, as follows: tertile I, <312 pg/mL (n = 89); tertile II, 312–441 pg/mL (n = 89); tertile III, >441 pg/mL (n = 89). The odds ratios were evaluated on multivariate logistic regression analysis. Model 1 included age, eGFR, and activin A levels. Model 2 included age, eGFR, hemoglobin levels, Mehran risk score, LVEF, and activin A levels. Model 3 included age, eGFR, hemoglobin levels, Mehran risk score, LVEF, smoking status, hypertension, diuretics use, proteinuria status, and activin A levels. CAG, coronary angiography; eGFR, estimated glomerular filtration rate; LVEF, left ventricular ejection fraction.

To evaluate the usefulness of pre-procedural serum activin A levels as biomarkers of CIN, we employed three multivariate logistic regression models and Tertile I was used as reference. All the variates that reach the statistically significant difference in Table 1 were integrated into the regression models. Model 1 included the covariates of age, eGFR, and activin A levels. Model 2 included the covariates of age, eGFR, hemoglobin levels, Mehran risk score, LVEF, and activin A levels. Model 3 included the covariates of age, eGFR, hemoglobin levels, Mehran risk score, LVEF, smoking status, hypertension, diuretics use, proteinuria status, and activin A levels. All three models revealed tertile III was significantly associated to incidence of CIN (versus tertile I), while tertile II did not exhibit significantly higher risk (Fig. 3).

**Figure 4:**
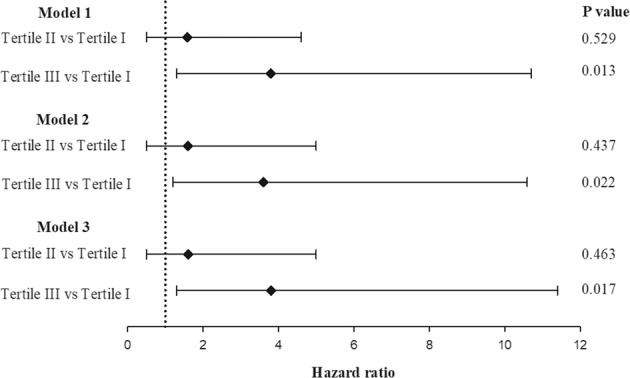
Predictive capability of pre-CAG levels of serum activin A for the risk of progressive renal function decline after CAG. The patients were stratified according to the tertiles of the distribution of activin A levels, as follows: tertile I, <312 pg/mL (n = 89); tertile II, 312–441 pg/mL (n = 89); tertile III, >441 pg/mL (n = 89). The hazard ratios were evaluated on multivariate Cox proportional regression analysis. Model 1 included age, eGFR, and activin A levels. Model 2 included age, eGFR, hemoglobin levels, Mehran risk score, LVEF, and activin A levels. Model 3 included age, eGFR, hemoglobin levels, Mehran risk score, LVEF, smoking status, hypertension, diuretics use, proteinuria status, CIN occurrence, and activin A levels. CAG, coronary angiography; eGFR, estimated glomerular filtration rate; LVEF, left ventricular ejection fraction; CIN, contract-induced nephropathy.

To evaluate the usefulness of serum activin A levels as biomarkers of progressive renal function decline after CAG, we employed three multivariate Cox proportional regression models based on the same predictor variables as described above for the logistic regression models, except for that the occurrence of CIN was integrated into model 3 to test whether serum activin A independently predicts long-term renal function decline. All three models highlighted tertile III were significantly associated to progressive renal function decline (versus tertile I), while tertile II did not exhibit significantly higher risk (Fig. 4). These findings indicated that serum activin A levels>441 pg/mL pre-CAG independently predict renal function decline after CAG.

## Discussion

The findings of this single-center study suggest that high serum activin A levels are associated with a higher risk of adverse renal outcomes after CAG, including immediate CIN and long-term progressive renal function decline. In addition, a pre-CAG serum activin A concentration>441 pg/mL independently predicts adverse renal outcomes. Thus, pre-CAG serum activin A levels may be considered to be integrated into CIN prevention strategies in future.

The association between activin A and kidney disease is well known. Animal models of kidney injury induced by ischemia/reperfusion^24,25^ or unilateral ureter obstruction^34^ revealed increased expression of activin A in the injured kidney. A mouse model of CKD revealed upregulation of activin expression in the skeleton, vasculature, heart, and kidneys^23^. In addition, a ligand trap for the activin type IIA receptor exhibited beneficial effects in murine CKD models, alleviating vascular disease and renal fibrosis^35^, renal osteodystrophy^36^ and nephrogenic anemia^37^. These previous findings implied a causative role of activin A in the pathogenesis of CKD and its potential role as a target of treatment. Taken together with our present observations, these previous findings support the rationale that high pre-CAG serum activin A levels may reflect minor pre-existing renal impairment and thus serve as predictors of renal outcomes after CAG.

Few clinical studies have examined the association between activin A and kidney disease. Urinary activin A was recently suggested to be a biomarker of AKI severity in humans^38^. In this previous study, urinary activin A was undetectable in healthy volunteers and patients with pre-renal AKI, but significantly increased in patients with established AKI, with poor correlation between serum and urinary activin A levels. On the contrary, our findings found the predictive value of serum activin A levels in the absence of kidney injury. Taken together, these findings suggested that serum and urinary activin A may have different and perhaps complementary clinical utility as biomarkers in kidney disease.

Several studies had shown increased serum activin A levels in cardiovascular disease. In patients with type 2 diabetes, serum activin A levels were associated with higher incidence of cardiovascular diseases^39,40^. A comprehensive study by Yndestad *et al*. found increased serum activin A levels in patients with CHF, concluding that abundant activin A may contribute to the pathogenesis of myocardial remodeling^27^. Furthermore, it has become widely accepted that heart failure leads to kidney injury or progressive renal function decline^5^. Considering the role of activin A in CHF, it may be an appropriate to use activin A as a biomarker of CIN risk in patients indicated for CAG, who generally have substantial prevalence of coronary artery disease and CHF.

Mehran risk score, which includes hypotension, intra-aortic balloon pump use, CHF, age >75 years, anemia, diabetes, contrast media volume, and eGFR as parameters, has been currently accepted as the most reliable prediction tool for CIN after CAG^10^. However, while the Mehran risk score has high prediction capability, its 8-item design is relatively complex, which limits its clinical application. In the present study, we showed that pre-CAG serum activin A may be a single, reliable biomarker for the prediction of CIN. The use of such single biomarker for CIN risk stratification may be more convenient and simple for clinical practice, and increase the awareness of the prevention of CIN.

A major limitation of the present study was that part of the participants whom revealed normal CAG results could not adhere the study protocol to follow up renal function in consecutive at least 1 year, which resulted in information bias. In addition, the number of the participants and follow-up events were relatively small, while most risk factors for CIN were considered in the multivariate analysis, we still could not control all possible confounding factors in order to avoid overfitting, which limited the statistical strength of this study. Another limitation was the single ethnicity of the present study, which limited the extension of the conclusion to other ethnic groups. Finally, while all dialysis events occurred in tertile III during follow-up period, the absent dialysis event in tertile I and II made it not feasible to perform multivariate regression using dialysis events as the outcome. On the other hand, the strength of the present study included comprehensive profile of CIN risk factors, relative long follow-up period, and the accurate documentation of outcome variables. In conclusion, our findings suggested the association between pre-CAG serum activin A levels and both CIN and progressive renal function decline. Additionally, we propose that serum activin A levels >441 pg/mL may be used as a biomarker of adverse renal outcomes.

## Supplementary information

Supplementary tables.
