# Author Correction: Serum Activin A Levels and Renal Outcomes After Coronary Angiography

**DOI:** 10.1038/s41598-020-63082-9

**Published:** 2020-04-10

**Authors:** Yi-Lin Tsai, Ruey-Hsing Chou, Ya-Wen Lu, Chung-te Liu, Po-Hsun Huang, Shing-Jong Lin

**Affiliations:** 1 Division of Cardiology, Department of Medicine, Taipei Veterans General Hospital, Taipei, Taiwan; 2 Cardiovascular Research Center, National Yang-Ming University, Taipei, Taiwan; 3 Department of Critical Care Medicine, Taipei Veterans General Hospital, Taipei, Taiwan; 4 Institute of Clinical Medicine, National Yang-Ming University, Taipei, Taiwan; 5 Healthcare and Management Center, Taipei Veterans General Hospital, Taipei, Taiwan; 6 Division of Nephrology, Department of Medicine, Wan Fang Hospital, Taipei Medical University, Taipei, Taiwan; 7 School of Medicine, College of Medicine, Taipei Medical University, Taipei, Taiwan; 8 Graduate Institute of Clinical Medicine, College of Medicine, Taipei Medical University, Taipei, Taiwan; 9 Taipei Heart Institute, Taipei Medical University Taipei, Taiwan

Correction to: *Scientific Reports* 10.1038/s41598-020-60359-x, published online 25 February 2020

In the original version of this Article, there were errors in Affiliations 2 and 9 which were incorrectly listed as ‘Cardiovascular Research Center, Taipei Veterans General Hospital, Taipei, Taiwan’ and ‘Board of Directors, Taipei Medical University, Taipei, Taiwan’, respectively.

The correct affiliations are listed below.

Affiliation 2

Cardiovascular Research Center, National Yang-Ming University, Taipei, Taiwan

Affiliation 9

Taipei Heart Institute, Taipei Medical University, Taipei, Taiwan

These errors have now been corrected in the PDF and HTML versions of the Article, and in the accompanying Supplemental Material.

