# Supplementary material for: Serum Activin A Levels and Renal Outcomes After Coronary Angiography
Source: Sci Rep. 2020 Feb 25;10:3365. doi: 10.1038/s41598-020-60359-x (PMC7042345; doi:10.1038/s41598-020-60359-x)
Supplement: Supplementary file 1 — Supplementary tables. [file 41598_2020_60359_MOESM1_ESM.docx]

**Serum Activin A Levels and Renal Outcomes After Coronary Angiography**

Yi-Lin Tsai, MD^1,2^, Ruey-Hsing Chou, MD^1,2,3,4^, Ya-Wen Lu, MD^1,2^, Chung-te Liu, MD^6,7,8#^, Po-Hsun Huang, MD, PhD^1,2,3,4^*^#^, Shing-Jong Lin, MD, PhD^1,2,4,5,9^

^1^Division of Cardiology, Department of Medicine, Taipei Veterans General Hospital, Taipei, Taiwan

^2^Cardiovascular Research Center, Taipei Veterans General Hospital, Taipei, Taiwan

^3^Department of Critical Care Medicine, Taipei Veterans General Hospital, Taipei, Taiwan

^4^Institute of Clinical Medicine, National Yang-Ming University, Taipei, Taiwan

^5^Healthcare and Management Center, Taipei Veterans General Hospital, Taipei, Taiwan

^6^Division of Nephrology, Department of Medicine, Wan Fang Hospital, Taipei Medical University, Taipei, Taiwan

^7^School of Medicine, College of Medicine, Taipei Medical University, Taipei, Taiwan

^8^Graduate Institute of Clinical Medicine, College of Medicine, Taipei Medical University, Taipei, Taiwan

^9^Board of Directors, Taipei Medical University, Taipei, Taiwan

**^#^Equal contribution**

***Corresponding author:** Professor Po-Hsun Huang, MD, PhD. Division of Cardiology, Department of Medicine, Taipei Veterans General Hospital, 112, No. 201, Sec. 2, Shih-Pai Road, Taipei, Taiwan. Tel: [+886-938590796](tel:%2B886-938590796). Fax: [+886-2-28763336](tel:%2B886-2-28763336). E-mail: [huangbsvgh@gmail.com](mailto:huangbsvgh@gmail.com)

**Running title:** Activin A and kidney injury after CAG

**Keywords:** activin A, contrast-induced nephropathy, acute kidney injury, chronic kidney disease

**Word count of abstract:** 199 words

**Word count of main text:** 2664 words

**Number of tables:** 3

**Number of figures:** 4

**Number of references:** 40

**Number of supplemental tables:** 2

| **Supplemental Table 1. Risk for CIN by univariate logistic regression** | | | |
| --- | --- | --- | --- |
| **Variable** | **OR** | **95% CI** | **P value** |
| Activin A tertile III | 3.97 | 1.39-11.27 | 0.010 |
| Age | 1.04 | 1.01-1.08 | 0.025 |
| Sex | 0.49 | 0.22-1.09 | 0.081 |
| BMI | 1.02 | 0.93-1.12 | 0.700 |
| Smoking | 1.02 | 0.45-2.32 | 0.970 |
| HTN | 1.53 | 0.56-4.22 | 0.409 |
| Type 2 DM | 2.24 | 1.00-4.99 | 0.049 |
| CHF | 2.24 | 0.91-5.48 | 0.078 |
| CAD | 1.23 | 0.89-1.71 | 0.210 |
| ACEi or ARB | 1.87 | 0.83-4.19 | 0.130 |
| Diuretics | 1.80 | 0.71-4.56 | 0.212 |
| Statin | 0.94 | 0.36-2.44 | 0.897 |
| Creatinine | 1.11 | 0.91-1.36 | 0.320 |
| Proteinuria | 2.70 | 1.15-6.29 | 0.022 |
| Hemoglobin | 0.66 | 0.53-0.82 | <0.001 |
| Fasting glucose | 1.01 | 0.99-1.01 | 0.104 |
| Uric acid | 0.97 | 0.79-1.18 | 0.747 |
| LVEF | 0.82 | 0.02-34.3 | 0.919 |
| Contrast volume | 1.00 | 0.99-1.01 | 0.240 |
| Mehran risk score | 1.14 | 1.06-1.23 | 0.001 |

CIN, contrast induced nephropathy; OR, odds ratio; CI, confidence interval; BMI, body mass index; HTN, hypertension; DM, Diabetes mellitus; CHF, congestive heart failure; CAD, coronary artery disease; ACEi, angiotensin converting enzyme inhibitor; ARB, angiotensin II receptor blocker; LVEF, left ventricular ejection fraction.

| **Supplemental Table 2. Risk for progressive renal function decline by univariate Cox proportional regression** | | | |
| --- | --- | --- | --- |
| **Variable** | **HR** | **95% CI** | ***p* value** |
| Activin A tertile III | 2.81 | 1.77-4.42 | <0.001 |
| Age | 0.99 | 0.98-1.02 | 0.876 |
| Sex | 0.45 | 0.25-0.81 | 0.007 |
| BMI | 0.93 | 0.86-1.01 | 0.090 |
| Smoking | 1.28 | 0.70-2.34 | 0.415 |
| HTN | 2.12 | 0.89-5.01 | 0.088 |
| Type 2 DM | 2.32 | 1.28-4.20 | 0.005 |
| CHF | 1.43 | 0.69-2.99 | 0.336 |
| CAD | 1.38 | 1.05-1.80 | 0.019 |
| ACEi or ARB | 2.26 | 1.24-4.12 | 0.007 |
| Diuretics | 2.30 | 1.22-4.36 | 0.011 |
| Statin | 1.38 | 0.72-2.63 | 0.336 |
| Creatinine | 1.24 | 1.15-1.35 | <0.001 |
| Proteinuria | 3.43 | 1.90-6.22 | <0.001 |
| Hemoglobin | 0.63 | 0.54-0.74 | <0.001 |
| Fasting glucose | 1.01 | 1.00-1.02 | 0.001 |
| Uric acid | 1.00 | 0.86-1.17 | 0.970 |
| LVEF | 0.08 | 0.01-0.97 | 0.047 |
| Contrast volume | 1.00 | 0.99-1.01 | 0.143 |
| Mehran risk score | 1.14 | 1.01-1.20 | <0.001 |

HR, Hazard ratio; CI, confidence interval; BMI, body mass index; HTN, hypertension; DM, Diabetes mellitus; CHF, congestive heart failure; CAD, coronary artery disease; ACEi, angiotensin converting enzyme inhibitor; ARB, angiotensin II receptor blocker; LVEF, left ventricular ejection fraction.
